# Immediate Ecological Impacts of the 2011 Tohoku Earthquake Tsunami on Intertidal Flat Communities

**DOI:** 10.1371/journal.pone.0062779

**Published:** 2013-05-01

**Authors:** Jotaro Urabe, Takao Suzuki, Tatsuki Nishita, Wataru Makino

**Affiliations:** Division of Ecology and Evolutionary Biology, Graduate School of Life Sciences, Tohoku University, Sendai, Japan; Bangor University, United Kingdom

## Abstract

Following the Great East Japan Earthquake in 2011, a large tsunami developed and struck the Pacific coast of eastern Japan. To assess the immediate impacts of the tsunami on coastal communities, changes in taxon composition and richness of macrobenthic animals before and after the tsunami were examined at nine intertidal flats in Sendai Bay and the Sanriku Ria coast. The results showed that 30–80% of taxa indigenously inhabiting intertidal flats disappeared after the tsunami. Among animal types, endobenthic and sessile epibenthic animals were more vulnerable to the tsunami than mobile epibenthic animals like shore crabs and snails. For all the intertidal flats examined, animals that were originally dwellers in lower tidal zones and not recorded before the tsunami were also found right after the tsunami, indicating that the tsunami not only took away many benthic taxa from the intertidal flats but also brought in some taxa from elsewhere. However, overall changes in taxon community composition were greater for intertidal flats that experienced larger inundation heights. These results showed that the ecological impacts of the tsunami were proportional to the physical impacts as gauged by wave height and that mobile epibenthic animals were less vulnerable to the tsunami.

## Introduction

Rare and extreme events such as unusual floods, hurricanes, droughts and oil spills often reduce abundance of major native species and enhance colonization success of minor native species and even exotic species [1]–[5]. These events may, therefore, become unique opportunities causing drastic changes in community structures and providing insights on ecological processes shaping community structures. However, due to limited opportunities, little general knowledge has been accumulated on the ecological impacts of these events [1]. To assess ecological impacts of rare and extreme events, we need to examine changes in community composition immediately after an event as a first and essential step.

Tsunami is one type of a rare and extreme event. Since they are natural disturbances that have repeatedly affected coastal ecosystems over long time scales, tsunamis may play a role in the formation of coastal communities [6], [7]. However, their ecological impacts are not, as yet, well understood. Using rare opportunities when tsunamis were generated by earthquakes, several studies have described subsequent structural changes of benthic communities in various coastal ecosystems including mangrove swamps [8], seagrass beds [9], sandy beaches [10] and subtidal soft bottoms [11]. These studies showed that the immediate impacts of a tsunami differed among communities at different sites and even among different species in the same ecosystem. However, it is not clear if the ecological impacts of a tsunami depend on the physical scale of the tsunami waves at a given site and what types of benthic animals are affected by tsunamis.

Following the Great East Japan Earthquake on 11 March 2011 registering 9.0 on the Moment Magnitude Scale (M_w_), the resulting large tsunami (the 2011 Tohoku earthquake tsunami) struck the Pacific coastline of eastern Japan, including Sendai Bay and the Sanriku Ria coast [12]. In these areas, a number of intertidal flats were found in lagoons of river mouths, in inner parts of the bay and on shallow sheltered beaches. The physical, social and economic impacts of the tsunami were documented [12]–[14]. However, few studies have examined the ecological impacts of the tsunami on intertidal flat communities. The exception was a study by Kanaya et al. [15] who examined changes in macrobenthic fauna on the Gamo intertidal flat of Sendai Bay after the tsunami. They showed that 30 out of 79 benthic species recorded on the flat for 7 years before the tsunami were not present after the tsunami.

However, it was not clear that such a change in benthic fauna was also induced by the 2011 Tohoku earthquake tsunami at other intertidal flats. Fortunately, we had made benthic fauna census surveys of several of the intertidal flats in Sendai Bay and on the Sanriku Ria coast before the tsunami. If the tsunami seriously affected the intertidal communities, taxon composition and richness of benthic animals should have changed. Moreover, the differences that occurred in benthic communities were likely larger at sites that received the greater physical impacts. We examined these possibilities by performing census surveys of the intertidal flats right after the tsunami at locations where surveys were made before the tsunami. Using the data from these surveys, we also examined what types of benthic animals were more affected by the tsunami.

## Methods

### Census Sites and Ethics Statement

**Figure 1:**
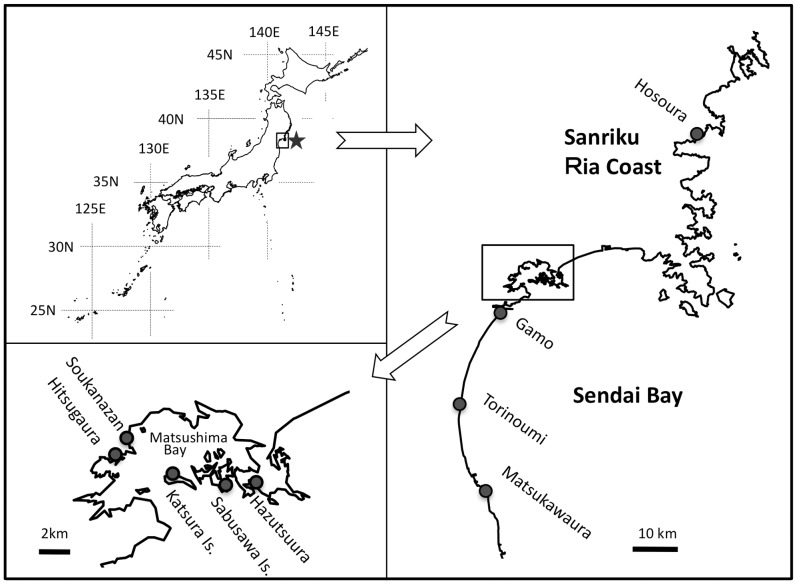
Maps of the Japanese Islands and the Pacific coastline of eastern Japan showing Sendai Bay and Matsushima Bay (an inner bay of Sendai Bay) with locations of the intertidal flats examined in this study (dark circles). Location of the seismic center of the Great East Japan Earthquake on 11 March 2011 is denoted by a star.

Pre-surveys of the areas subjected to the 2011 Tohoku earthquake tsunami were conducted in March to June between 2002 and 2010 and post-surveys in May to August 2011 at nine intertidal flats in Sendai Bay and on the Sanriku Ria coast (Table. 1, Fig. 1). All of these intertidal flats are so-called muddy flats and consisted of silt, clays and fine sands, that are exposed at low tide. The amount of exposed area differed among the intertidal flats and varied from 1 to 350 ha. Among these, Matsukawaura and Torinoumi, the two largest flats, and Gamo are located in coastal lagoons. Hitsugaura, Sokanzan and Hazutsuura are located on the shore of Matsushima Bay, while Katsura and Sabusawa are located on sheltered beaches of small islands in Matsushima Bay. Hosoura occurs on the Sanriku Ria coast. Because these intertidal flats are not privately owned or legislatively protected, no special permits were required to perform field surveys in these areas. No protected species were involved in this study.

### Census Method

Taxon richness and composition of macrobenthos on intertidal flats were examined during low tide using intertidal census survey methods described below. Since surveys were done by the naked eye, animals recorded were those larger than ca. 5 mm in body size. Identification was generally made on-site using live animals. These animals were released after identification for nature conservation purpose. However, for animal taxa that could not be visually identified, several individuals were fixed with formalin and identified microscopically in the laboratory. Identification was generally made to species level. For animals that could not be identified to species level, identification was made to genus or the finest taxonomic level possible. Throughout the censuses, the same researcher confirmed taxon identifications to minimize artifacts caused by misidentification. The identified animals were categorized into three different types according to mobility and habitat: i.e., sessile epibenthic animals (SA) that lived by attaching to substrates at the sediment surface, mobile epibenthic animals (MA) that were able to move on the sediment surface, and endobenthic animas (EA) that lived in sediments. Because not all animals were identified to the species level, community characteristics were expressed as taxon richness and composition. In this study, taxon richness was defined simply as number of taxa found in a single survey.

Since the surveys before the tsunami were originally done for different objects, the census was made in different methods and date from spring to summer depending on sites. Fortunately, in some cases, different census methods were performed at the same date or site (Table 1). Therefore, census method and season were included in statistical analyses examining effects of tsunami on taxon richness. In sum, for the nine intertidal flats, total 11 census surveys were done before the tsunami and 13 surveys after the tsunami. In each survey, one of the following three census methods was used.

**Table 1:** Longitude and latitude (expressed in the decimal system) of intertidal flats examined, and date and census methods of the surveys before and after the 2011 Tohoku earthquake tsunami.

				Before the tsunami		After the tsunami	
Site	Latitude	Longitude	Surface area	Date	CensusMethod	Date	Census Method
Hosoura	38.69516	141.49884	2 ha	17-May-10	M1	11-Jun-11	M1
Hazutsuura	38.33216	141.14649	6 ha	28-May-02	M2	20-May-11	M1
Sabusawa Is.	38.33580	141.08732	1 ha	24-May-04	M1	18-Jun-11	M1
Katsura Is.	38.33001	141.12644	1 ha	16-Mar-09	M1	13-Aug-11	M3
Hitsugaura	38.35066	141.05203	4 ha	28-May-02	M2	28-May-11	M2
				28-Apr-09	M1	03-Jun-11	M3
Soukanzan	38.35253	141.05943	2 ha	08-Jun-09	M1	03-Jun-11	M2
						03-Jun-11	M3
Gamo	38.25574	141.01248	3 ha	22-May-04	M2	31-Jul-11	M3
						01-Aug-11	M2
Torinoumi	38.03096	140.91330	60 ha	05-Jun-08	M3	16-Jun-11	M3
				05-Jun-08	M3	16-Jun-11	M3
Matsukawaura	37.81933	140.98217	350 ha	04-Jun-08	M3	05-Jun-11	M3

In Method 1 (M1), one to three field researchers walked randomly for 30 to 90 min over whole area of a small intertidal flat or over an area <1 ha of large intertidal flats, and searched for epibenthic animals. Researchers often dug holes of approximately 30-cm in diameter and about 20 cm in depth with shovels and visually searched for endobenthic animals in muddy sediments. The animals found were either recorded to the finest taxonomic level and returned to the intertidal flat or were collected for later microscopic identification in the laboratory.

In Method 2 (M2), at a given intertidal flat, three transect lines, from the shore at low water level to the land, were first set. In each transect, a 5×5 m quadrant was placed near shore, midpoint and near land. Thus, a total of nine quadrants (total 0.023 ha) covering most of an intertidal flat was established. Animals found in the quadrants were collected and identified. In each quadrant, the substrate (about 20 cm in depth) was removed for 10 min by shovel and endobenthic animals were examined as in Method 1. The animals found were either recorded to the finest taxonomic level or collected for later identification as above.

In Method 3 (M3), eight to twelve researchers randomly walked for 15 min over the entire area of a given intertidal flat, and collected epibenthic animals they encountered. Each researcher also dug 15 holes of approximately 30-cm in diameter and about 20 cm in depth with shovels and visually searched endobenthic animals. The animals easily identified on-site were returned to the intertidal flat after recording, but those unidentified were taken to the laboratory for further examination as above. This method has been described in detail elsewhere [16].

### Statistical Analyses

To examine differences in taxon richness before and after the tsunami, a generalized linear mixed model (GLMM) was used with the model selection procedure employing Akaike’s information criteria (AIC) [17]. In the model for taxon richness, tsunami (pre-tsunami and post-tsunami surveys), season (surveys in March-May, June and July-August) and census methods (methods 1, 2 and 3) were treated as fixed effects, and intertidal flat as a random effect. The model assumed Poisson distribution and all of the explanatory variables were used as categorical data. Because the surveys were made only once in March and August, these were grouped with April-May and July, respectively. The intertidal flats examined differed greatly in surface area and location (Fig. 1, Table 1). In addition, texture, particle composition and nutrient condition of the sediments probably differed among the intertidal flats. To control for the possible influence of these site-specific conditions on taxon richness, intertidal flat was incorporated in the models as a random effect. Models were constructed separately for sessile epibenthic animals (SA), mobile epibenthic animals (MA), endobenthic animals (EA) and all animals (AA). For each animal category, a model with the lowest AIC value and models with an AIC not greater than 2 from the lowest AIC were selected as the best models [17].

Similarity (S) in taxon composition before and after the tsunami was measured by Jaccard index [18] as follows,
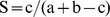
where a and b are taxon richness before and after the tsunami, respectively, and c is number of taxa commonly found both before and after the tsunami. Percent changes in number of taxa (T), percent taxa remaining (R) and percent new taxa (N) were calculated as follows:



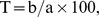


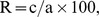


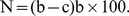



If differences in taxon richness and taxonomic composition were caused mainly by the tsunami, values of these parameters were likely related to the physical impacts of the tsunami. To test this possibility, the relations of S, T, R and N with inundation height of the tsunami at each intertidal flat were examined by simple correlation analyses. The inundation height was obtained from data compiled by the 2011 Tohoku earthquake tsunami joint survey group [12] (http://www.coastal.jp/tsunami2011/). For intertidal flats where no inundation height was recorded, an average value of inundation heights (two to four) at coastal sites within 1 km radius was used.

## Results and Discussion

**Figure 2:**
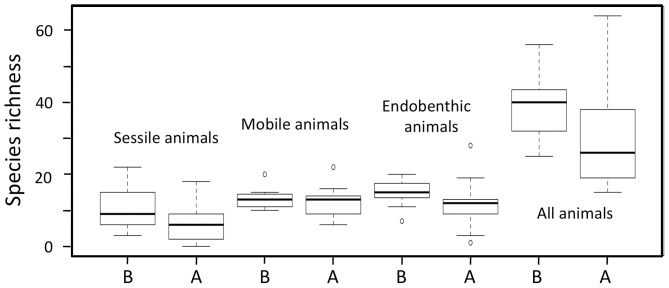
Box plots showing taxon richness of sessile epibenthic animals, mobile epibenthic animals, endobenthic animals and all animals before (B) and just after the tsunami (A).

Before the tsunami struck in 2011, a total of 172 benthic animal taxa (mainly at the species level) were recorded from nine intertidal flats (Table S1). After the tsunami, a total of 140 animal taxa were found at the same set of intertidal flats. Among these, the number of taxa commonly found both before and after the tsunami was only 106. Thus, 66 taxa were not found in the surveys immediately following the tsunami and 34 taxa were benthic animals newly recorded for the areas. On average, animal taxon richness per intertidal flat decreased by 22% after the tsunami (Fig. 2).

To clarify if the difference in number of benthic animal taxon richness before and after the tsunami was due to either differences in the census methods used or seasons when the surveys were performed or both, a GLMM was constructed with season, census method and tsunami as fixed factors and intertidal flat as a random factor. The best model for taxon richness of all benthic animals (AA) included only tsunami (timing of survey) as an explanatory variable (Table 2). The result suggested that the difference in taxon richness between pre-tsunami and post-tsunami surveys was mainly caused by the tsunami and not attributable to either census methods or seasons.

**Table 2:** Results from the GLMM analyses showing the best models for taxon richness of sessile epibenthic animals (SA), mobile epibenthic animals (MA), endobenthic animals (EA) and all animals (AA) with fixed effects for census methods (M2 and M3), tsunami (T) and their interactions.

No.	Model		df	AIC	Chi-square	P-value
1	SA∼M2+M3+T+(1|Site)		5	62.06	20.07	<0.001
2	MA∼(1|Site)		2	30.05		
3	MA∼T+(1|Site)		3	31.71	0.76	0.384
4	EA∼M2+M3+T+M2*T+M3*T+(1|Site)		7	47.02	29.37	<0.001
5	AA∼T+(1|Site)		3	69.39	12.16	<0.001
**No.**	**Intercept**	**Coefficient of M2**	**Coefficient of M3**	**Coefficient of T**	Coefficient of T*M2	Coefficient of T*M3
1	2.608+0.165	−0.687+0.226	−0.573+0.230	−0.314+0.160		
2	2.509+0.078					
3	2.563+0.098			−0.101+0.116		
4	2.602+0.149	0.227+0.217	0.217+0.242	−1.247+0.314	1.153+0.402	1.115+0.379
5	3.660+0.086			−0.245+0.070		

The models used site as a random effect (1|Site) controlling for the possible influence of site-specific conditions. Results of Chi-square tests against the null model and coefficients of the best models with the standard deviations are shown.

Among animals, sessile epibenthic animals and endobenthic animals decreased in taxon richness in surveys after the tsunami (Fig. 2). To explore whether these changes were attributable to the tsunami, a GLMM was also constructed for each of these types of animals. The best models for sessile and endobenthic animals included tsunami as an explanatory variable, although some of the changes were attributable to differences in census method, as shown by inclusion of census method in the best models (Table 2). For mobile epibenthic animals, differences in taxon richness were small between surveys before and after the tsunami, and only one of the two best models included tsunami as an explanatory variable. The results indicated that compared with sessile animals such as chitons (*Patelloida conulus*) and sponges (*Halichondria panicea*) and endobenthic animals such as bivalves (*Laternula marilina* and *Macoma incongrua*) and polychaetes (species of Nereididae), mobile epibenthic animals like shore crabs (*Hemigrapsus penicillatus, H. takanoi*) and hermit crab*s* (*Pagurus minutus*) were less affected by the tsunami.

**Figure 3:**
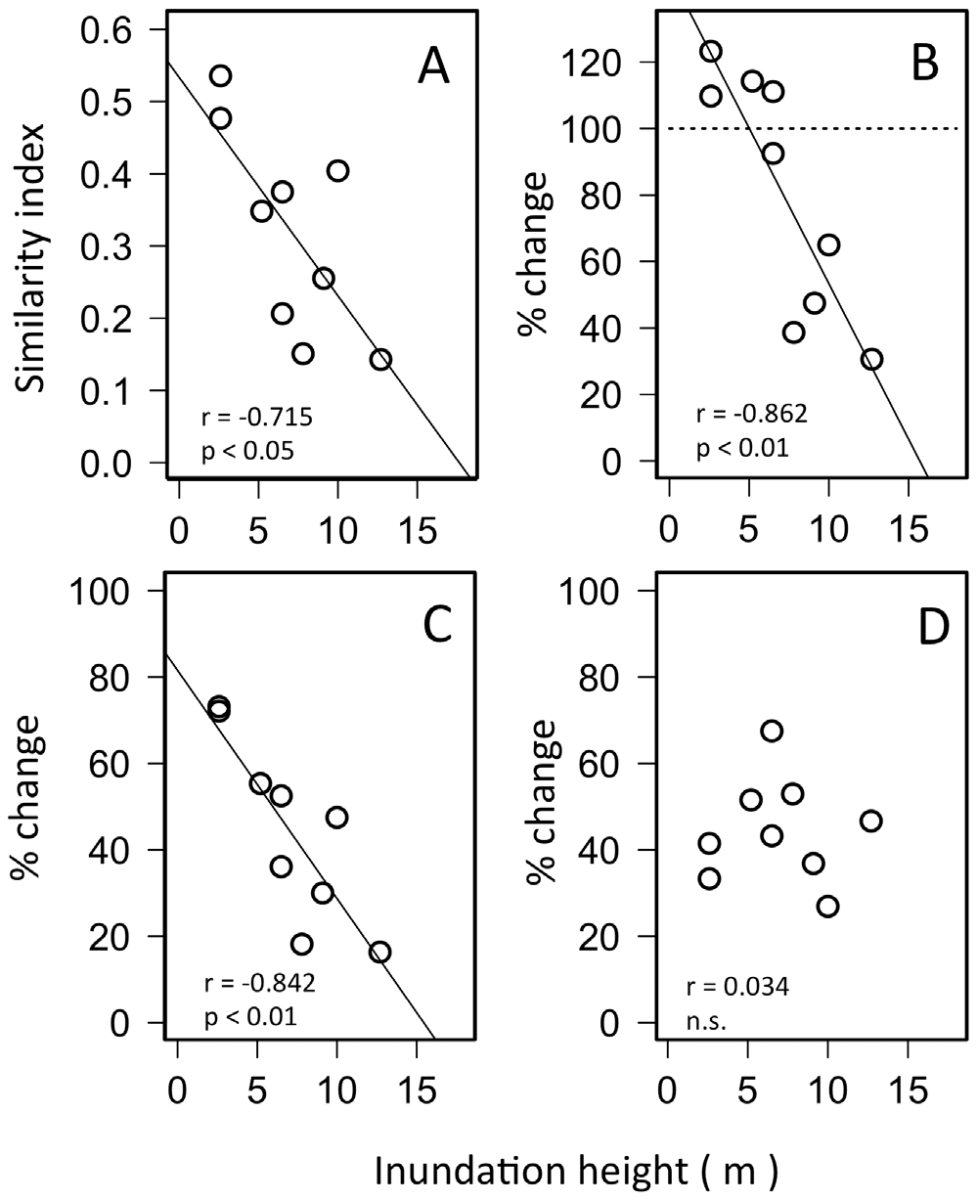
Jaccard similarity (see text) in taxon composition before and after the tsunami (A), percent changes in number of taxa (B), percent taxa remaining (C) and percent new taxa (D) plotted against inundation height of the tsunami. Correlation coefficients with significance levels are shown in each panel.

Because pre-tsunami surveys were made in different years among the sites, one may suspect that changes in taxon composition and richness between pre-tsunami and post-tsunami surveys reflect a simple annual variation. However, if these changes were indeed caused by the 2011 Tohoku earthquake tsunami, it was most likely that the changes were greater for intertidal flats receiving larger tsunami waves. One method to gauge the magnitude of physical impacts brought on by the tsunami is to use inundation height. Due to the local geomorphology, the height differed among sites and location on the Pacific coastline of eastern Japan [12]; it was <3 m for intertidal flats located at inner parts of Sendai Bay but >9 m for intertidal flats formed in lagoons that faced the Pacific Ocean. To examine effects of physical impacts by the tsunami, community composition similarity between pre- and post-tsunami at each flat are plotted against inundation height (Fig. 3a). It decreased significantly for intertidal flats having larger inundation heights. Significant correlation to inundation height was also detected for percent changes in number of taxa and percent taxa remaining. For some intertidal flats facing the Pacific Ocean (Matsukawaura, Hosoura and Sabusawa Is.), the similarity value was <0.2 and community composition changed drastically after the tsunami. At these intertidal flats, the number of taxa was halved by the tsunami and percent taxa remaining was <20%. However, more than 70% of taxa survived at intertidal flats in inner parts of Sendai Bay (Soukanzan and Hitsugaura) where the inundation height was <3 m (Fig 3b).

These results clearly indicated that taxon composition and richness of intertidal flat communities in Sendai Bay and on the Sanriku Ria coast were mainly changed by the 2011 Tohoku earthquake tsunami and that these changes were greater for intertidal flats that experienced tsunami waves with larger inundation heights. The fact implies that ecological impacts of the tsunami were proportional to the physical impacts as gauged by wave height. Because of their rare and extreme nature, only a limited number of studies have assessed immediate impacts of tsunamis on benthic communities [8]–[11]. Comparison of these studies suggests that ecological impacts of tsunamis vary depending on physical impacts. For example, with the 2007 Peru earthquake (M_W_ 8.0), taxon composition of the benthic community of the soft bottom beach of Paracas Bay was largely unchanged after a tsunami with a run-up height of 2–3 m, although the abundance of several species were changed [11]. In general, run-up height of a tsunami is larger than the inundation height used in the present study. Thus, physical impacts of the tsunami reported for the beach of Paracas Bay would be much smaller than those observed in our study. Whanpetch et al. [9] examined impacts of the tsunami generated by the Sumatra–Andaman earthquake (M_w_ 9.1–9.3) in 2004 on seagrass beds and benthic communities therein and showed that changes in community structures after the tsunami were larger at the site facing open ocean, which likely experienced larger physical forces of the tsunami waves than more sheltered sites. This study showed that inundation or wave height is a useful gauge when immediate impacts of tsunamis on benthic communities are to be compared across different sites and events.

In the present study, taxon richness of mobile epibenthic animals was less affected by the tsunami compared to sessile and endobenthic animals. With the 2010 Chilean earthquake (M_W_ 8.8), abundance of mobile crustacean species on some sandy beaches was either unaffected or increased within two months of the tsunami [10]. Similarly, in mangrove swamps on the Indian Ocean coast of Thailand, abundance of endobenthic animals, like bivalves, decreased drastically after the tsunami generated by the Sumatra–Andaman earthquake (M_w_ 9.1–9.3) but those of mobile epibenthic animals, like crustaceans and snails, did not [8]. The results of these previous studies [8], 10 are consistent with those of our study, implying that mobile epibenthic animals have abilities to persist against large physical disturbances or return promptly within a few months from unknown refuge habitats.

In estuaries with muddy bottoms, opportunistic polychaeta often increase in abundance right after large disturbances. In the Neuse River Estuary of North Carolina, for example, an opportunistic polychaete species showed a notable increase in abundance within two months following extensive damage by multiple hurricanes in 1999, although other native macrobenthic abundance declined. Similarly, Kanaya et al. [15] found that the abundance of endobenthic polycheates, such as *Hediste diadroma* and *Pseudopolydora* cf. *kempi*, increased on the Gamo intertidal flat five months after the 2011 Tohoku earthquake tsunami. These are known to be opportunistic species [19]. We also found these opportunistic polychaetes at several but not all intertidal flats examined after the tsunami. This implies that physical disturbance induced by the 2011 Tohoku erathquack tsunami favored opportunistic species at some intertidal flats, including Gamo.

Worth noting from these observation is that the effects of tsunamis are not limited to a species “take-away” action from intertidal flats. After the tsunami struck, animals that were not recorded before the tsunami were found at all the intertidal flats. For intertidal flats in the inner part of Sendai Bay, the number of taxa after the tsunami was larger than before the tsunami (>100%: Fig. 3B). The percentage of new taxa was not correlated with tsunami inundation height (Fig. 3D). These animals included species of sea cucumber (*Stichopus japonica*), sea star (*Asterina pectinifera*) and swimming crab (*Charybdis japonica*). Because most of these were originally dwellers in lower tidal zones, they were temporally brought onto the intertidal flats by the tsunami. We expect that these animal taxa will disappear from the intertidal flats shortly.

Recent studies suggest that large, rare and extreme events like unusual floods, hurricanes and tsunamis change the physical and chemical conditions of habitats [2], [3], [4], [11], [15], [20] and may provide an opportunity for invasive species to colonize areas by transporting their propagules and depressing the abundance of native species [1]. Moreover, the events may push a community to an alternative state with a different taxon composition [21]. Large, rare and extreme events, therefore, are likely important processes in shaping and altering local community structures [1], [2]. This implies that events like a tsunami generate not only immediate impacts but also long-lasting impacts on local communities. Among animal taxa recorded in the pre-tsunami surveys of the intertidal flats examined, 66 taxa were not found in the post-tsunami surveys. These animals might have been present in low numbers and been underway in re-establishing their populations. However, the tsunami may have created habitat conditions that impaired recovery of some native species. If this was the case, the 2011 Tohoku earthquake tsunami could result in long-lasting impacts on the intertidal flat communities of Sendai Bay and the Sanriku Ria coast. Long-term monitoring is essential to examine community-level recovery processes and thus to understand implications of tsunamis on coastal ecosystems.

## Supporting Information

Table S1 **Taxon list and results of census surveys used in this study.**
(PDF)
