# Correction: Immediate Ecological Impacts of the 2011 Tohoku Earthquake Tsunami on Intertidal Flat Communities

**DOI:** 10.1371/annotation/61f59d8c-7ee1-4288-8571-16a00321c92a

**Published:** 2013-10-17

**Authors:** Jotaro Urabe, Takao Suzuki, Tatsuki Nishita, Wataru Makino

An error was introduced in the preparation of this article for publication. In the Methods section, there is an error in the fourth equation of the "Statistical Analyses" section. There is a "/" missing between (b-c) and b. The correct equation is N=(b-c)/b x 100. 

