# Supplementary material for: Immediate Ecological Impacts of the 2011 Tohoku Earthquake Tsunami on Intertidal Flat Communities
Source: PLoS One. 2013 May 1;8(5):e62779. doi: 10.1371/journal.pone.0062779 (PMC3641098; doi:10.1371/journal.pone.0062779)
Supplement: Table S1 — Taxon list and results of census surveys used in this study. (PDF) [file pone.0062779.s001.pdf]

Table S1. Taxon list and results of census surveys used in this study. Species found in each census survey are denoted by "1". Life forms are denoted by s (sessile epibenthic animal), m (mobile epibenthic animal), and e (endobenthic animal).

			Before the Tsunami											After the Tsunami												
	Site		Hosoura	Hazutsuura	Sabusawa Is.	Katsurajima Is	Hitsugaura	Hitugaura	Soukanzan	Gamo Higata	Torinoumi(1)	Trinoumi(2)	Matsukawaura	Hosoura	Hazutsuura	Sabusawa Is.	Katsurajima Is	Hitsugaura	Hitsugaura	Soukanzan	Soukanzan	Gamo Higata	Gamo Higata	Torinoumi(1)	Trinoumi(2)	Matsukawaura
	Date		2010.5.17	2002.5.28	2004.5.24	2009.3.16	2002.5.28	2009.4.28	2009.6.8	2004.5.22	2008.6.5	2008.6.5	2008.6.4	2011.6.11	2011.5.20	2011.6.18	2011.8.13	2011.5.28	2011.6.3	2011.6.3	2011.6.3	2011.7.31	2011.8.1	2011.6.16	2011.6.16	2011.6.5
	Census method		1	2	1	1	2	1	1	2	3	3	3	1	1	1	3	2	3	2	3	3	2	3	3	3
	Species name	Life form																								
1	*Acanthochitona achates*	s	0	1	0	1	0	0	1	0	0	0	1	0	1	0	1	0	0	1	1	0	0	0	0	0
2	*Acanthopleura* sp.	s	1	0	0	1	0	0	0	0	0	0	0	0	1	0	0	0	0	0	1	0	0	0	0	0
3	Actiniaria	s	1	0	0	0	0	0	0	1	0	0	1	0	0	0	0	0	0	0	0	0	0	0	0	0
4	*Alpheus brevicristatus*	e	0	1	0	1	0	0	1	0	1	1	0	0	0	0	1	1	0	0	0	0	0	0	0	0
5	*Amaroucium pliciferum*	s	0	0	0	1	0	0	0	0	0	0	0	0	0	0	0	0	0	0	0	0	0	0	0	0
6	Ampithoe	m	0	0	0	1	0	0	0	0	0	0	0	1	0	0	1	0	0	0	0	0	0	1	1	1
7	Angustassiminea	m	0	0	0	1	0	1	0	0	0	0	0	0	0	0	0	0	0	0	0	0	0	0	0	0
8	*Angustassiminea castanea*	m	0	0	0	0	0	0	0	1	0	0	0	0	0	0	0	1	0	0	0	1	0	0	0	0
9	*Angustassiminea parasitologica*	m	0	0	0	0	0	0	0	1	0	0	0	0	0	0	0	0	0	0	0	0	0	0	0	0
10	*Angustassiminea yoshidayukioi*	m	0	0	0	1	0	0	0	0	0	0	0	0	0	0	0	0	0	0	0	0	0	0	0	0
11	*Anthopleura fuscoviridis*	s	0	0	1	0	0	0	0	0	0	0	0	0	0	0	0	0	0	0	0	0	0	0	0	0
12	*Anthopleura kurogane*	s	1	0	0	0	0	0	0	0	0	0	0	0	0	0	0	0	0	0	0	0	0	0	0	0
13	*Anthopleura uchidai*	s	0	0	0	0	0	0	0	0	0	0	0	0	1	0	0	0	0	0	0	0	0	0	0	0
14	*Apocepon pulcher*	s	0	0	0	0	0	0	0	0	0	0	0	0	0	0	1	0	0	0	0	0	0	0	0	0
15	*Apohyale barbicornis*	m	1	0	0	0	0	0	0	0	0	0	0	0	0	0	0	0	0	0	0	0	0	0	0	0
16	*Arenicola brasiliensis*	e	0	0	1	0	0	0	0	0	0	0	1	0	0	0	1	0	0	0	0	0	0	0	0	1
17	*Armandia lanceolata*	m	0	1	0	0	0	0	0	0	0	0	0	0	0	0	0	0	0	0	0	0	0	0	0	0
18	Ascidiacea	s	0	0	0	0	0	0	0	0	0	0	0	0	0	0	1	0	0	0	0	0	0	0	0	0
19	Assiminea	m	0	1	0	0	0	0	1	0	0	0	1	0	0	0	0	0	0	0	0	0	0	0	0	0
20	Assiminea	m	0	1	0	0	0	0	0	0	0	0	0	0	1	0	0	0	0	0	0	0	0	0	0	0
21	*Assiminea estuarina*	m	0	0	0	0	0	1	0	0	0	0	0	0	0	0	0	0	0	0	0	0	0	0	0	0
22	*Assiminea hiradoensis*	m	0	0	0	0	0	1	0	1	0	1	0	0	0	0	0	0	0	0	0	1	1	1	0	0
23	*Assiminia japonica*	m	0	0	0	0	0	0	0	0	0	0	0	0	0	0	0	0	0	0	0	0	0	1	0	0
24	*Asterina pectinifera*	m	0	0	0	0	0	0	0	0	0	0	0	0	1	0	0	0	0	0	0	0	0	0	0	0
25	Balanina	s	0	0	1	0	0	0	0	0	0	0	0	0	0	0	0	0	0	0	0	0	0	0	0	0
26	*Balanus albicostatus*	s	0	1	0	1	0	1	1	1	1	1	1	0	1	1	1	1	1	1	0	0	0	0	0	0
27	*Balanus glandula*	s	1	0	0	0	0	0	0	0	0	0	0	1	0	0	0	0	0	0	0	0	0	0	0	0
28	*Balanus improvisus*	s	0	0	0	0	0	0	0	0	0	0	0	0	0	1	1	0	0	0	0	0	0	0	0	0
29	*Barnea manilensis*	e	0	0	1	1	0	0	0	0	0	0	0	0	0	0	1	0	0	0	0	0	0	0	0	0
30	*Batillaria attramentaria*	m	0	0	1	1	1	1	1	0	1	1	1	0	1	0	1	1	1	1	1	0	0	1	1	1
31	*Batillaria multiformis*	m	0	0	1	1	1	1	1	1	0	1	1	0	1	0	0	1	1	1	1	0	0	0	0	0
32	*Batillaria zonalis*	m	0	1	0	0	0	0	0	0	0	0	0	0	1	0	0	0	0	0	0	0	0	0	0	0
33	Botrylloides	s	0	0	0	1	0	0	0	0	0	0	1	0	0	0	0	0	0	0	0	0	0	0	0	1
34	*Camptandrium sexdentatum*	m	0	0	0	0	0	0	0	0	0	0	0	0	0	0	0	0	0	0	1	0	0	0	0	0
35	Capitellidae	e	0	0	0	0	0	0	0	0	0	0	0	0	0	0	0	0	0	0	1	0	1	0	0	0
36	Capitllidae	e	0	0	0	0	0	0	0	1	1	1	1	0	0	0	0	0	0	0	0	1	1	0	0	0
37	*Cecina manchurica*	m	0	0	0	1	0	0	0	0	0	0	0	0	0	0	0	0	0	0	0	0	0	0	0	0
38	*Ceratonereis erythraeensis*	e	0	0	0	1	1	0	1	0	0	0	0	0	0	0	1	1	0	1	1	0	0	0	0	0
39	*Ceratostoma inornatus*	s	0	0	0	1	0	0	0	0	0	0	0	0	0	0	1	0	0	0	0	0	0	0	0	0
40	Cerebratulidae	e	0	1	0	0	0	0	0	0	0	0	0	0	0	0	0	0	0	0	0	0	0	0	0	0
41	*Cerebratulus communis*	e	0	0	0	0	0	0	0	1	1	1	0	0	0	0	1	0	0	1	0	1	1	0	1	0
42	*Cerithidea djadjariensis*	m	0	0	0	0	1	1	0	0	0	0	0	0	0	0	0	1	1	0	0	0	0	0	0	0
43	*Cerithidea rhizophorarum*	m	0	0	0	0	1	1	0	1	0	0	0	0	0	0	0	1	0	0	0	1	1	0	0	0
44	*Chaetopterus variopedatus*	e	0	1	0	0	0	0	0	0	0	0	0	0	0	0	0	0	0	0	0	0	0	0	0	0
45	*Charybdis japonica*	m	0	0	0	0	0	0	0	0	0	0	0	0	0	0	1	0	0	0	0	0	0	0	0	0
46	*Chiromantes haematocheir*	e	0	0	0	0	0	1	0	0	0	0	0	0	0	0	0	0	0	0	0	0	0	0	0	0
47	*Chrolostoma lischkei*	s	1	0	1	0	0	0	0	0	0	0	0	0	1	0	0	0	0	0	0	0	0	0	0	0
48	*Chthamalus challengeri*	s	0	0	0	0	0	0	1	0	0	0	0	0	0	0	0	0	0	0	0	0	0	0	0	0
49	*Cirriformia tentaculata*	e	0	1	1	0	1	0	1	0	1	1	1	0	1	0	1	1	1	1	0	0	0	1	0	0
50	*Cleantiella isopus*	m	0	0	0	0	0	0	0	0	0	0	0	0	0	0	0	0	0	0	1	0	0	0	0	0
51	*Collisella* sp.	s	0	0	1	0	0	0	0	0	0	0	0	0	0	0	0	0	0	0	0	0	0	0	0	0
52	Corophioidea	e	0	0	0	0	0	0	0	0	0	0	1	0	0	1	1	0	0	0	0	0	0	0	0	1
53	*Crangon* sp.	m	0	0	0	0	0	0	0	0	1	1	0	0	0	0	0	0	0	0	1	0	0	0	0	0
54	*Crassostrea gigas*	s	1	1	1	1	1	1	1	1	1	1	1	0	1	1	1	1	1	1	1	0	0	0	1	0
55	*Crepidula onyx*	s	0	0	0	1	0	0	0	0	0	0	1	0	0	0	0	0	0	0	0	0	0	0	0	0
56	Cryptocelididae	m	0	1	0	0	0	0	0	0	0	0	0	0	0	0	0	0	0	0	0	0	0	0	0	0
57	Cyathura	e	0	0	0	0	0	0	0	0	1	0	0	0	0	0	0	0	0	0	0	0	0	0	1	0
58	*Cyclina sinensis*	e	0	0	0	0	1	1	1	0	0	0	1	0	1	0	1	1	1	1	1	0	0	0	0	0
59	*Cymodoce japonica*	m	0	0	1	0	0	0	0	0	0	0	0	0	0	0	0	0	0	0	0	0	0	0	0	0
60	*Deiratonotus cristatus*	e	0	0	0	0	0	0	0	1	0	1	0	0	0	0	0	0	0	0	0	1	1	1	0	0
61	*Dendostrea paulucciae*	s	0	0	1	0	0	0	0	0	0	0	0	0	0	0	0	0	0	0	0	0	0	0	0	0

(Table S3. continue)

			Before the Tsunami											Before the Tsunami												
	Site		Hosoura	Hazutsuura	Sabusawa Is.	Katsurajima Is	Hitsugaura	Hitugaura	Soukanzan	Gamo Higata	Torinoumi(1)	Trinoumi(2)	Matsukawaura	Hosoura	Hazutsuura	Sabusawa Is.	Katsurajima Is	Hitsugaura	Hitsugaura	Soukanzan	Soukanzan	Gamo Higata	Gamo Higata	Torinoumi(1)	Trinoumi(2)	Matsukawaura
	Date		2010.5.17	2002.5.28	2004.5.24	2009.3.16	2002.5.28	2009.4.28	2009.6.8	2004.5.22	2008.6.5	2008.6.5	2008.6.4	2011.6.11	2011.5.20	2011.6.18	2011.8.13	2011.5.28	2011.6.3	2011.6.3	2011.6.3	2011.7.31	2011.8.1	2011.6.16	2011.6.16	2011.6.5
	Census method		1	2	1	1	2	1	1	2	3	3	3	1	1	1	3	2	3	2	3	3	2	3	3	3
	Species name	Life form																								
62	Diopatra sugokai	e	1	0	1	1	0	0	0	0	0	0	0	0	1	0	1	0	0	1	1	0	0	0	0	0
63	Eogammarus possjeticus	m	0	0	0	0	0	0	0	0	0	0	0	0	0	0	1	0	0	1	1	0	0	1	1	0
64	Euspira fortunei	m	0	0	0	1	0	0	1	0	1	1	1	0	0	0	1	1	1	1	0	0	0	0	1	0
65	Eutaeniichthys gilli	m	0	0	0	0	0	0	0	1	0	0	0	0	0	0	0	0	0	0	0	0	0	0	1	0
66	Gaetice depressus	m	0	0	1	0	0	0	0	0	0	0	0	0	1	1	0	0	0	0	0	0	0	0	0	0
67	Gammaridea	m	0	0	0	0	0	0	0	0	1	1	0	0	1	0	0	0	0	0	0	1	0	0	0	0
68	Glossaulax didyma	m	1	0	0	0	0	0	0	0	0	0	0	0	0	0	0	0	0	0	0	0	0	0	0	0
69	Glycera pacifica	e	0	1	0	0	0	0	0	0	0	0	0	0	0	0	0	0	0	0	0	0	0	0	0	0
70	Glyceridae	e	0	0	1	0	0	0	0	0	1	0	1	0	0	0	0	0	0	0	0	0	0	0	0	0
71	Gnorymosphaeroma sp.	m	0	0	0	1	0	0	0	0	1	0	0	0	0	0	0	0	0	0	0	0	0	1	0	0
72	Gobioidei	m	0	0	0	0	0	0	0	0	0	0	0	0	0	0	0	0	0	0	0	0	0	0	0	0
73	Gomphina melanegis	e	0	0	0	0	0	0	0	0	0	0	0	0	0	0	0	0	0	0	0	1	1	0	0	0
74	Goniadidae	e	0	0	0	0	0	0	0	0	0	0	0	0	0	0	0	0	0	0	0	0	0	0	0	1
75	grandidierella japonica	e	0	0	0	0	0	0	0	0	0	0	0	0	0	0	1	0	1	0	1	0	0	1	1	1
76	Gymnogobius macrognathos	m	0	0	0	0	0	0	0	1	0	0	0	0	0	0	0	0	0	0	0	0	0	0	1	0
77	Gyractis japonica	s	1	0	0	1	0	0	0	0	0	0	0	0	0	0	1	0	0	0	0	0	0	0	0	0
78	Hadiste japonica	e	0	0	0	0	1	0	1	0	0	0	0	0	0	0	0	0	1	0	0	0	0	0	0	0
79	Halichondria japonica	s	0	0	0	0	0	0	1	0	0	0	0	0	0	0	0	0	0	0	0	0	0	0	0	0
80	Halichondria panicea	s	0	0	0	1	0	0	1	0	0	0	0	0	0	0	0	0	0	0	0	0	0	0	0	0
81	Haliclona permollis	s	0	0	0	1	0	0	0	0	0	0	0	0	0	0	0	0	0	0	0	0	0	0	0	0
82	Haliplanella lineata	s	0	0	1	1	1	0	1	1	0	0	0	0	1	1	1	1	1	1	1	0	0	1	0	0
83	Hediste atoka	e	0	0	0	0	0	0	0	0	0	0	0	0	0	0	0	0	0	0	0	0	0	1	0	0
84	Hediste diadroma	e	0	0	0	0	0	0	0	1	0	0	0	0	0	0	0	0	0	0	0	1	1	1	1	0
85	Helice tridens	m	0	0	0	0	1	1	0	1	0	1	1	0	0	0	0	1	1	0	0	1	1	1	0	0
86	Hemigrapsus penicillatus	m	1	0	0	0	1	1	1	0	1	1	1	1	1	0	1	1	1	1	1	1	0	1	1	1
87	Hemigrapsus sanguineus	m	1	0	1	0	0	0	1	0	0	0	0	1	1	1	1	1	1	0	0	1	0	0	0	0
88	Hemigrapsus takanoi	m	1	0	0	1	0	0	1	0	0	0	1	0	0	1	1	1	1	0	1	0	0	1	1	1
89	Heteromastus sp.	e	1	1	0	0	1	0	0	0	0	0	0	0	1	0	0	1	1	0	0	0	0	0	0	0
90	Hippolytidae	m	0	0	0	1	0	0	0	0	0	0	0	0	0	0	0	0	0	0	0	0	0	0	0	0
91	Holometopus dehaani	e	0	0	0	0	0	0	0	1	0	0	0	0	0	0	0	0	0	0	0	1	0	0	0	0
92	Hormomya mutabilis	s	0	0	0	1	0	0	0	0	0	0	0	0	0	0	0	0	0	0	0	0	0	0	0	0
93	Hyale sp.	m	0	0	0	0	0	0	0	0	0	0	1	0	0	0	1	0	0	0	0	0	0	0	0	1
94	Idotea ochotensis	m	0	0	0	0	0	0	0	0	0	0	0	0	0	0	1	1	0	0	0	0	0	0	0	0
95	Ilyoplax pusilla	m	0	0	0	0	1	1	0	1	0	0	1	0	0	0	0	0	1	0	0	1	1	0	0	0
96	Jesogammarus	m	0	0	0	0	0	0	0	0	0	0	0	0	0	1	0	0	0	0	0	0	0	0	0	0
97	Laemodonta exaratoides	s	0	0	0	1	0	1	0	0	0	0	0	0	0	0	0	1	0	0	0	0	0	0	0	0
98	Laomedia astacina	e	0	0	0	0	1	1	1	1	0	0	0	0	0	0	0	0	1	1	1	0	0	0	0	0
99	Laternula (Exolaternula) marilina	e	0	1	0	0	1	0	1	1	1	1	1	0	0	0	0	0	0	0	0	0	0	0	0	0
100	Leitoscoloplos pugettensis	e	1	0	1	0	0	0	0	0	0	0	0	0	0	0	0	0	0	0	0	0	0	0	0	0
101	Lepidonotus	m	0	1	0	0	0	0	0	0	0	0	0	0	0	0	0	0	0	0	0	0	0	0	0	0
102	Ligia cinerascens	m	0	0	0	1	0	1	1	1	0	0	1	1	1	0	1	1	1	1	1	1	1	0	0	0
103	Ligia exotica	m	0	0	0	0	0	0	0	0	0	0	0	0	0	1	0	0	0	0	0	0	0	0	0	0
104	Limbrineris nipponica	e	1	1	0	0	1	0	1	0	1	1	1	0	1	0	1	1	0	1	1	0	0	1	1	0
105	Littorina brevicula	s	1	0	1	1	0	1	1	0	1	0	1	1	1	1	0	1	0	1	1	0	0	0	0	0
106	Logis bocki	e	0	0	1	0	0	0	0	0	0	0	0	0	0	0	0	1	0	0	1	0	0	0	0	0
107	Lottia	s	1	0	0	0	0	0	0	0	0	0	0	0	0	0	0	0	0	0	0	0	0	0	0	0
108	Luciogobius guttatus	m	1	0	0	0	0	0	0	0	0	0	0	0	0	0	1	0	0	0	0	0	0	1	0	0
109	Lumbrineridae	e	1	0	0	0	0	0	0	0	0	0	0	0	0	0	0	0	0	0	0	0	0	0	0	0
110	Macoma (Macoma) contabulata	e	0	0	0	0	0	0	0	1	0	1	0	0	0	0	0	1	0	0	0	1	1	1	1	1
111	Macoma incongrua	e	1	0	1	0	0	0	1	0	1	1	1	0	1	0	0	1	1	1	1	0	0	0	0	0
112	Macrophthalmus japonicus	m	0	0	0	0	1	1	1	1	1	1	1	0	0	0	0	1	1	0	1	0	0	1	1	1
113	Mactra chinensis	e	1	0	0	1	0	0	0	0	0	0	0	0	0	0	0	0	0	0	0	0	0	0	0	0
114	Maldanidae	e	1	0	0	0	0	0	0	0	0	0	0	0	0	0	0	0	0	0	0	0	0	0	0	0
115	Marphysa sanguinea	e	0	1	0	1	0	0	1	0	0	0	0	0	0	0	0	1	0	1	0	0	0	0	0	0
116	Melanochlamys	m	0	0	0	0	0	0	0	0	1	0	0	0	0	0	0	0	0	0	0	0	0	0	0	0
117	Melita setiflagella	m	0	0	0	1	0	0	0	0	0	0	0	0	0	0	1	0	1	1	0	0	0	1	1	0
118	Melita shimizui	m	1	0	0	0	0	0	0	0	0	0	0	0	0	0	0	0	1	0	0	0	0	0	0	0
119	Meretrix lusoria	e	0	1	0	1	0	0	0	0	1	0	0	0	0	0	0	0	0	0	0	0	0	0	0	0
120	Metabopyrus ovalis	s	0	0	0	0	0	0	0	1	0	0	0	0	0	0	0	0	0	0	0	0	0	0	0	0
121	Modiolus nipponicus	s	0	0	0	0	0	0	0	0	0	0	0	0	1	0	0	0	0	0	0	0	0	0	0	0
122	Moerella rutila	e	1	0	0	0	0	0	1	0	0	0	0	0	0	0	1	1	1	1	1	0	0	0	0	0
123	Monodonta labio	s	1	0	1	0	0	1	1	0	0	1	0	1	1	0	0	1	1	1	0	0	0	0	0	0
124	Monodonta neritoides	s	1	0	0	0	0	0	0	0	0	0	0	0	0	0	0	0	0	0	0	0	0	0	0	0
125	Musculista senhousia	s	0	0	1	1	0	0	0	0	0	1	1	0	0	0	0	0	0	0	0	0	0	0	0	0

(Table S3. continue)

	Species name	Life form	Before the Tsunami											Before the Tsunami												
	Site		Hosoura	Hazutsuura	Sabusawa Is.	Katsurajima Is	Hitsugaura	Hitugaura	Soukanzan	Gamo Higata	Torinoumi(1)	Trinoumi(2)	Matsukawaura	Hosoura	Hazutsuura	Sabusawa Is.	Katsurajima Is	Hitsugaura	Hitsugaura	Soukanzan	Soukanzan	Gamo Higata	Gamo Higata	Torinoumi(1)	Trinoumi(2)	Matsukawaura
	Date		2010.5.17	2002.5.28	2004.5.24	2009.3.16	2002.5.28	2009.4.28	2009.6.8	2004.5.22	2008.6.5	2008.6.5	2008.6.4	2011.6.11	2011.5.20	2011.6.18	2011.8.13	2011.5.28	2011.6.3	2011.6.3	2011.6.3	2011.7.31	2011.8.1	2011.6.16	2011.6.16	2011.6.5
	Census method		1	2	1	1	2	1	1	2	3	3	3	1	1	1	3	2	3	2	3	3	2	3	3	3
126	*Mya(Arenomya)arenaria oonogai*	e	0	0	0	0	0	0	0	0	0	0	0	0	0	0	1	0	0	1	0	0	0	0	1	0
127	*Mytilus galloprovincialis*	s	1	0	1	1	0	1	1	0	1	1	1	1	1	1	1	1	1	1	0	1	0	1	0	1
128	*Neanthes virens*	e	1	0	0	0	0	0	0	0	0	0	0	0	0	0	0	0	0	0	0	0	0	0	0	0
129	*Nectoneanthes latipoda*	e	0	0	0	0	0	0	0	0	0	0	0	0	0	0	1	0	0	1	0	0	0	0	0	0
130	Nemertea	e	1	0	1	0	0	0	0	0	0	0	0	0	0	0	1	0	0	0	0	0	0	0	0	0
131	*Neomysis awatschensis*	m	0	0	0	0	1	0	0	1	0	0	0	0	0	0	0	0	0	0	0	0	0	0	0	0
132	*Neomysis* sp.	m	1	0	0	0	0	0	0	0	0	0	0	0	0	0	0	0	0	0	0	0	0	0	0	0
133	Nephtyoidea	e	1	0	0	0	0	0	0	0	0	0	0	0	0	0	1	0	0	0	0	0	0	0	0	0
134	*Nephtys caeca*	e	0	1	0	0	0	0	0	0	0	0	0	0	0	0	0	0	0	0	0	0	0	0	0	0
135	*Nephtys polybranchia*	m	0	1	0	0	0	0	0	0	0	0	0	0	0	0	0	0	0	0	0	0	0	0	0	0
136	Nereididae	e	0	1	1	0	0	0	0	0	1	1	1	0	0	0	0	0	0	0	0	0	0	0	0	0
137	*Nereis vexillosa*	e	0	0	0	0	0	0	0	0	0	0	0	1	0	0	0	0	0	0	0	0	0	0	0	0
138	*Nihonotrypaea harmandi*	e	0	0	1	0	0	0	0	0	0	0	0	0	0	0	0	0	0	0	0	0	0	0	0	0
139	*Nihonotrypaea japonica*	e	1	1	0	0	0	0	1	1	1	1	0	0	0	0	0	0	0	1	1	0	0	0	1	0
140	Nipponacmea	s	1	0	0	0	0	0	0	0	0	0	0	1	1	0	0	0	0	0	0	0	0	0	0	0
141	*Nipponophyllum japonicum*	m	0	0	0	0	0	0	0	0	0	0	0	0	0	0	1	0	0	0	0	0	0	0	0	0
142	*Nitidotellina nitidula*	e	1	0	0	0	0	0	0	0	0	0	0	0	0	0	0	0	0	0	0	0	0	0	0	0
143	*Nodilittorina radiata*	s	0	0	0	0	0	0	1	0	0	0	0	0	0	0	0	0	0	1	0	0	0	0	0	0
144	*Notoacmea concinna*	s	0	0	0	1	0	0	1	0	0	0	0	0	0	1	0	0	0	0	0	0	0	0	0	0
145	*Notoacmea teramachii*	s	0	0	0	0	0	0	0	0	0	0	0	0	0	1	0	0	0	0	0	0	0	0	0	0
146	*Notomastus* sp.	e	0	0	1	0	0	0	0	1	0	0	0	0	0	0	1	0	0	0	0	0	0	0	0	0
147	*Nuttallia olivacea*	e	0	0	0	0	1	0	1	1	1	1	1	0	0	0	0	0	0	1	1	1	1	1	1	0
148	Nynantheae	s	0	1	0	0	0	0	0	0	0	0	0	0	0	0	0	0	0	0	0	0	0	0	0	0
149	*Ocypode stimpsoni*	e	0	0	0	0	0	0	1	0	0	0	0	0	0	0	1	0	0	0	0	0	0	0	0	0
150	*Omphalius pfeifferi pfeifferi*	s	1	0	0	0	0	0	0	0	0	0	0	0	0	0	0	0	0	0	0	0	0	0	0	0
151	*Omphalius rusticus*	s	1	0	1	1	0	0	0	0	0	0	0	0	1	0	1	0	0	0	0	0	0	0	0	0
152	Ophiuroidea	m	0	0	1	0	0	0	0	0	0	0	0	0	0	0	0	0	0	0	1	0	0	0	0	0
153	*Pagurus minutus*	m	1	1	1	1	1	0	1	0	1	1	0	0	1	1	1	1	1	1	1	1	0	1	1	1
154	*Palaemon* sp.	m	1	0	0	1	0	0	0	0	0	0	1	0	0	0	1	0	0	0	0	0	0	0	1	1
155	*Pariambus* sp.	m	0	0	0	1	0	0	0	0	0	0	0	0	0	0	1	0	0	0	0	0	0	0	0	0
156	*Patelloida conulus*	s	0	1	0	1	0	0	0	0	0	0	1	0	0	0	0	0	0	0	0	0	0	0	0	0
157	*Patelloida pygmaea*	s	0	0	1	0	0	1	0	0	1	1	1	0	1	0	1	1	1	1	0	0	0	0	0	0
158	*Patelloida pygmaea*	s	0	0	0	0	0	0	0	0	0	0	0	0	0	1	0	0	0	0	0	0	0	0	0	0
159	*Perinereis muntia brevicirris*	e	1	0	1	0	0	1	1	1	0	0	0	1	1	0	0	1	1	1	1	0	0	0	0	0
160	*Phacosoma japonicum*	e	0	0	0	1	0	0	0	0	0	0	0	0	0	0	1	0	0	1	0	0	0	0	0	0
161	*Philyra pisum*	m	0	1	1	1	0	0	1	0	1	1	0	0	1	0	1	0	1	1	1	0	0	0	1	0
162	Phyllodocidae	e	0	0	0	0	0	0	0	0	0	0	0	0	0	0	1	0	0	0	1	0	0	0	0	0
163	*Pillucina pisidium*	e	0	0	0	0	0	0	0	0	0	0	0	0	0	0	1	0	0	0	0	0	0	0	0	0
164	*Platorchestia platensis*	m	0	0	0	0	0	1	0	1	0	1	0	0	0	0	1	0	0	0	0	1	1	0	0	0
165	Polycladida	m	1	0	0	1	0	0	0	0	0	0	0	0	0	0	1	0	0	1	0	0	0	0	0	0
166	Polydora sp.	m	0	0	0	0	0	0	0	1	0	0	0	0	0	0	0	0	0	0	0	0	0	0	0	0
167	Polynoidea	e	0	0	0	1	0	0	0	0	0	0	0	0	0	0	1	0	0	0	1	0	0	0	0	0
168	Pontodrilus	e	0	0	0	1	0	1	0	0	0	0	0	0	0	0	1	0	0	0	0	0	0	0	0	0
169	Porifera	s	1	0	0	0	0	0	0	0	0	0	0	0	0	0	0	0	0	0	0	0	0	0	0	0
170	*Portunus trituberculatus*	m	1	0	0	0	0	0	0	0	0	0	0	0	1	0	0	0	0	0	0	0	0	0	0	0
171	*prionospio japonica*	e	0	0	0	0	0	0	0	1	0	0	0	0	0	0	0	0	0	0	0	0	0	0	0	0
172	*Pseudocardium sachalinense*	e	0	0	1	0	0	0	0	0	0	0	0	0	0	0	0	0	0	0	0	0	0	0	0	0
173	*Pseudopolydora kempi*	e	1	0	0	0	0	0	0	1	0	0	0	0	0	0	0	0	0	0	0	1	1	1	1	0
174	*Pseudoscorpionida* sp.	m	0	0	0	1	0	0	0	0	0	0	0	0	0	0	0	0	0	0	0	0	0	0	0	0
175	*Reishia bronni*	s	0	0	1	0	0	0	0	0	0	0	0	0	1	0	0	0	0	0	0	0	0	0	0	0
176	*Reticunassa festiva*	m	1	1	1	0	1	1	1	0	1	1	1	0	0	0	1	0	1	1	1	0	0	0	0	0
177	*Reticunassa fratercula*	m	1	0	1	1	0	0	0	0	0	0	0	0	0	0	1	0	0	0	0	0	0	0	0	0
178	Rhizocephalan	s	0	0	0	0	0	0	0	0	0	0	0	0	0	0	1	0	0	0	0	0	0	0	0	0
179	*Ruditapes philippinarum*	e	1	1	1	1	1	0	1	1	1	1	0	0	1	0	1	1	1	1	1	1	1	0	1	0
180	sabellidae	e	0	0	0	0	0	0	0	1	0	0	0	0	0	0	0	0	0	0	0	0	0	0	0	0
181	*Scopimera globosa*	e	0	1	1	0	1	1	1	1	0	1	1	0	1	0	1	0	1	1	1	1	1	0	1	1
182	*Septifer virgatus*	s	0	0	0	0	0	0	0	0	0	0	0	1	1	0	0	0	0	0	0	0	0	0	0	0
183	Serpulidae	s	1	0	0	1	0	0	0	0	0	0	0	0	0	0	1	0	0	0	0	0	0	0	0	0
184	*Sestrostom toriumii*	e	0	1	1	0	0	0	0	0	0	0	0	0	0	0	0	0	0	0	0	0	0	0	0	0
185	*Siphonosoma cumanense*	e	0	1	0	0	0	0	0	0	0	0	0	0	0	0	0	0	0	0	0	0	0	0	0	0
186	*Solen strictus*	e	0	1	0	0	0	0	0	1	0	0	1	0	0	0	1	0	0	0	0	0	0	0	0	0
187	*Sphaeroma retrolaevis*	m	0	0	0	0	0	0	0	1	0	0	0	0	0	0	0	0	0	0	0	0	0	0	0	0
188	Sphaeromidae	m	1	0	0	0	0	0	0	0	0	0	1	0	0	0	0	0	0	0	0	0	0	0	0	0
189	*Sternaspis scutata*	e	0	0	0	0	0	0	0	0	0	0	0	0	0	0	0	0	0	0	1	0	0	0	0	0

(Table S3. continue)

	Species name	Life form	Before the Tsunami										
	Site		Hosoura	Hazutsuura	Sabusawa Is.	Katsurajima Is	Hitsugaura	Hitugaura	Soukanzan	Gamo Higata	Torinoumi(1)	Trinoumi(2)	Matsukawaura
	Date		2010.5.17	2002.5.28	2004.5.24	2009.3.16	2002.5.28	2009.4.28	2009.6.8	2004.5.22	2008.6.5	2008.6.5	2008.6.4
	Census method		1	2	1	1	2	1	1	2	3	3	3
190	*Stichopus japonica*	m	0	0	0	0	0	0	0	0	0	0	0
191	*Styela clava Herdman*	s	0	0	0	0	0	0	0	0	0	0	1
192	Syllidae	e	0	0	0	0	0	0	0	0	0	0	0
193	Talitridae	m	0	0	0	0	0	0	0	0	0	0	0
194	*Telmessus acutidens*	m	0	0	0	0	0	0	0	0	0	0	0
195	*Thais clavigera*	s	0	1	1	0	0	0	1	0	0	0	0
196	*Thelepus japonicus*	e	0	1	1	1	0	0	0	0	0	0	0
197	*Trapezium liratum*	s	0	0	0	0	1	1	1	0	0	0	0
198	*Truncatella pfeifferi*	m	0	0	0	1	0	0	0	0	0	0	0
199	Tubifex	e	0	0	0	0	0	0	0	1	0	0	0
200	*Turbo (Lunella) coreensis*	s	0	0	1	1	0	0	0	0	0	0	1
201	*Tylorrhynchus heterochaetus*	e	0	0	0	0	0	0	0	1	0	0	0
202	*Umbonium moniliferum*	e	0	0	0	1	0	0	0	0	0	0	0
203	*Upogebia major*	e	0	1	0	0	0	0	0	0	0	0	0
204	*Upogebia yokoyai*	e	0	0	0	0	0	1	0	0	1	0	0
205	*Urechis unicinctus*	e	0	0	0	1	0	0	0	0	0	0	0
206	*Xenostrobus atratus*	s	0	0	0	0	0	1	0	0	0	0	0

	Species name	Before the Tsunami												
	Site	Hosoura	Hazutsuura	Sabusawa Is.	Katsurajima Is	Hitsugaura	Hitsugaura	Soukanzan	Soukanzan	Gamo Higata	Gamo Higata	Torinoumi(1)	Trinoumi(2)	Matsukawaura
	Date	2011.6.11	2011.5.20	2011.6.18	2011.8.13	2011.5.28	2011.6.3	2011.6.3	2011.6.3	2011.7.31	2011.8.1	2011.6.16	2011.6.16	2011.6.5
	Census method	1	1	1	3	2	3	2	3	3	2	3	3	3
190	*Stichopus japonica*	0	0	0	0	0	0	0	0	0	0	0	0	1
191	*Styela clava Herdman*	0	0	0	0	0	0	0	0	0	0	0	0	1
192	Syllidae	0	0	0	0	0	0	1	0	0	0	0	0	0
193	Talitridae	1	0	0	0	0	0	0	0	0	0	0	0	0
194	*Telmessus acutidens*	1	0	0	0	0	0	0	0	0	0	0	0	0
195	*Thais clavigera*	0	1	1	0	0	0	0	0	0	0	0	0	0
196	*Thelepus japonicus*	1	0	0	1	0	0	0	0	0	0	0	0	0
197	*Trapezium liratum*	0	0	0	0	1	1	0	0	0	0	0	0	0
198	*Truncatella pfeifferi*	0	0	0	0	0	0	0	0	0	0	0	0	0
199	Tubifex	0	0	0	0	0	0	0	0	0	1	0	0	0
200	*Turbo (Lunella) coreensis*	0	1	0	0	0	0	0	0	0	0	0	0	0
201	*Tylorrhynchus heterochaetus*	0	0	0	0	0	0	0	0	1	1	1	0	0
202	*Umbonium moniliferum*	0	0	0	1	0	0	0	0	0	0	0	0	0
203	*Upogebia major*	0	0	0	0	0	0	0	0	0	0	0	0	0
204	*Upogebia yokoyai*	0	0	0	0	0	0	0	1	0	0	0	0	0
205	*Urechis unicinctus*	0	0	0	0	0	0	0	0	0	0	0	0	1
206	*Xenostrobus atratus*	0	0	0	0	0	0	0	0	0	0	0	0	0
